# Therapeutic response to leflunomide in combo therapy and monotherapy is associated to serum teriflunomide (A77 1726) levels

**DOI:** 10.1038/s41598-022-05644-7

**Published:** 2022-02-03

**Authors:** Nicte S. Fajardo-Robledo, Heriberto Jacobo-Cuevas, Edsaul E. Perez-Guerrero, Esther G. Corona-Sanchez, A. Miriam Saldaña-Cruz, Elba M. Romero-Tejeda, N. Alejandra Rodriguez-Jimenez, Sylvia E. Totsuka-Sutto, Rocio I. Lopez-Roa, Juan M. Ponce-Guarneros, M. Fabiola Alcaraz-Lopez, Sergio Cerpa-Cruz, J. Francisco Muñoz-Valle, E. German Cardona-Muñoz, Laura Gonzalez-Lopez, Jorge I. Gamez-Nava, Research Group for Factors Related to Therapeutic Outcomes in Autoimmune Diseases, Nicte Selene Fajardo-Robledo, Heriberto Jacobo-Cuevas, Ana Miriam Saldaña-Cruz, Norma Alejandra Rodriguez-Jimenez, Juan Manuel Ponce-Guarneros, Miriam Fabiola Alcaraz-Lopez, Ernesto German Cardona-Muñoz, Laura Gonzalez-Lopez, Jorge Ivan Gamez-Nava

**Affiliations:** 1 Laboratorio de Investigación y Desarrollo Farmacéutico, Centro Universitario de Ciencias Exactas e Ingenierías, Universidad de Guadalajara, 44430 Guadalajara, Jalisco Mexico; 2 Programa de Doctorado en Farmacología, Centro Universitario de Ciencias de la Salud, Universidad de Guadalajara, 44340 Guadalajara, Jalisco Mexico; 3 Centro Universitario de Ciencias de la Salud, Instituto de Investigación en Ciencias Biomédicas, Universidad de Guadalajara, 44340 Guadalajara, Jalisco Mexico; 4 Centro Universitario de Ciencias de la Salud, Instituto de Investigación en Reumatologia y del Sistema Musculo-Esqueletico, Universidad de Guadalajara, 44340 Guadalajara, Jalisco Mexico; 5 Departamento de Fisiología, Centro Universitario de Ciencias de la Salud, Universidad de Guadalajara, 44340 Guadalajara, Jalisco Mexico; 6 Departamento de Farmacobiologia, Centro Universitario de Ciencias Exactas e Ingenierías, Universidad de Guadalajara, 44430 Guadalajara, Jalisco Mexico; 7 Instituto Mexicano del Seguro Social, UMF 97, 46474 Magdalena, Jalisco Mexico; 8 Instituto Mexicano del Seguro Social, HGR 46, 44340 Guadalajara, Jalisco Mexico; 9 División de Reumatología, Hospital Civil de Guadalajara “Fray Antonio Alcalde”, Guadalajara, Jalisco México; 10 Programa de Doctorado en Salud Publica, Departamento de Salud Publica, Centro Universitario de Ciencias de la Salud, Universidad de Guadalajara, Guadalajara, Jalisco Mexico

**Keywords:** Biomarkers, Medical research, Rheumatology, Risk factors

## Abstract

There is a significant rate of therapeutic failure in rheumatoid arthritis (RA) patients treated with leflunomide (LEF). This study investigates the utility values of teriflunomide levels (A77 1726) in identifying RA patients who remained with moderate or severe disease activity after the treatment with LEF. In this cross-sectional study, we compared: (a) RA patients who achieved a DAS28-ESR ≤ 3.2, and (b) RA patients who maintained a DAS28-ESR > 3.2 after treatment. ROC curves determined the cut-off of A77 1726 with the better performance to identify patients achieving a DAS28-ESR ≤ 3.2. Of the 115 patients treated with LEF, 69 (60%) remained with moderate/severe disease activity and 46 (40%) achieved low disease activity/remission. Higher A77 1726 levels showed a negative correlation with DAS28-ESR (r = − 0.42, *p* < 0.001) and other parameters of disease activity. We obtained the following utility values with the cut-off of A77 1726 > 10 µg/mL to identify RA patients who achieved a DAS28-ESR ≤ 3.2: sensitivity of 91.31%; specificity of 73.91%; positive predictive value of 70.00%; and negative predictive value of 92.73%. Serum A77 1726 discriminated between RA patients who remained with moderate/severe disease activity despite the treatment with LEF both as monotherapy and LEF as combo therapy.

## Introduction

Rheumatoid arthritis (RA) is an autoimmune systemic disease characterized by chronic synovitis involving synovial joints that lead to joint destruction, functionality loss, and health-related quality-of-life impairment^1,2^. In Mexico, approximately 1.6% of the adult population is diagnosed with RA^3^. Clinical practice guidelines for the treatment of RA suggest the adoption of a conventional synthetic disease-modifying antirheumatic drug (csDMARD) as the first-line treatment^4–6^. Methotrexate (MTX) is the most frequently prescribed csDMARD as the first-line treatment for RA patients, although inefficacy, side effects or non-acceptance and withdrawal of this csDMARD can be usually observed^7^. Thus, other csDMARDs can be prescribed as either monotherapy or combo therapy. Leflunomide (LEF) is considered an alternative to MTX, and is for recommended for RA patients who fail to achieve an adequate response to MTX or when this drug is contraindicated^8,9^. LEF is also considered to increase efficacy as combo with other csDMARDs^4–6^.

Nevertheless, a high proportion of RA patients have a therapeutic failure to LEF. Strand et al. have reported that approximately 52% of RA patients present an inadequate response to LEF^10^; however, some factors related to the failure of LEF in RA are currently not well known. LEF administered orally at a standard dosage of 20 mg/day is metabolized by the cytochrome P450 2C19 enzyme (CYP2C19), which creates teriflunomide (A77 1726), an active metabolite of LEF^11^. Therefore, LEF is a prodrug that is rapidly converted to its active metabolite A77 1726/teriflunomide which has anti-rheumatic and immunomodulatory effects^12^. To date, there are a limited number of studies that assess the relationship between A77 1726 concentrations and therapeutic response to LEF administered as monotherapy in RA patients^13,14^; however, there are no studies that assess the value of teriflunomide levels in identifying those patients who achieved a DAS28-ESR ≤ 3.2 when treated with LEF as combo csDMARD therapy in RA patients. Some published studies performed in RA patients treated with LEF as monotherapy have described a relation between high A77 1726 concentrations and a decrease in the number of swollen joints^13^; or other therapeutic responses^14^. However, to our knowledge, no studies have assessed the significance of A77 1726 levels as a biomarker of therapeutic response in RA patients using LEF as combo therapy with other csDMARDs. Moreover, we have no information on the comparison of utility values (including sensitivity, specificity, and predictive values) of serum levels of A77 1726 between the monotherapy and combo therapy csDMARDs strategies. This information is required to identify whether the measurement of these levels can be useful in the clinical context to predict the therapeutic response in both strategies.

Therefore, the present study aimed to investigate the utility values of teriflunomide (A77 1726) levels in identifying rheumatoid arthritis (RA) patients who remained with moderate or severe disease activity after treatment with leflunomide (LEF), either as monotherapy or in combination with other disease-modifying antirheumatic drugs.

## Results

Table 1 presents a description of the clinical characteristics of 115 patients with RA that were included in this study. These patients had a mean age of 60 years, mean disease duration of 16 years, and a mean DAS28-ESR of 4.3. Of the 115 patients, 69 (60%) had moderate/severe disease activity after treatment, 46 (40%) had low disease activity/remission, and 47% of these patients were in LEF monotherapy.

**Table 1:** Clinical characteristics of RA patients included in the study.

Variable	n = 115
Age (years)	60 ± 11
RA disease duration (years)	16 ± 11
**Clinical characteristics**	
Swollen joints, mean ± SD	3.1 ± 3.4
**Response according DAS28-ESR achieved with leflunomide**	
Pt. who remained with a DAS28-ESR > 3.2 after treatment, n (%)	69 (60)
Pt. who achieved a DAS28-ESR ≤ 3.2), n (%)	46 (40)
**Treatment**	
Leflunomide, n (%)	115 (100)
Leflunomide monotherapy n (%)	54 (47.0)
Leflunomide combined with other DMARDs n (%)	61 (53.0)
NSAID’s user, n (%)	107 (93.0)
Corticosteroid users, n (%)	85 (73.0)
**Laboratory variables**	
C-reactive protein, (mg/L), mean ± SD	18 ± 23
Erythrocyte sedimentation rate, (mm/h), Mean ± SD	28 ± 12
Teriflunomide (A77 1726) levels, µg/mL	21.03 ± 28.62

Quantitative variables were expressed as the means and standard deviations and qualitative variables, as frequencies and percentages (%).

*RA* rheumatoid arthritis, *DAS28-ESR* disease activity score using erythrocyte sedimentation rate, *ESR* erythrocyte sedimentation rate, *Pt* patient, *NSAID’s* non-steroidal anti-inflammatory drugs.

**Figure 1:**
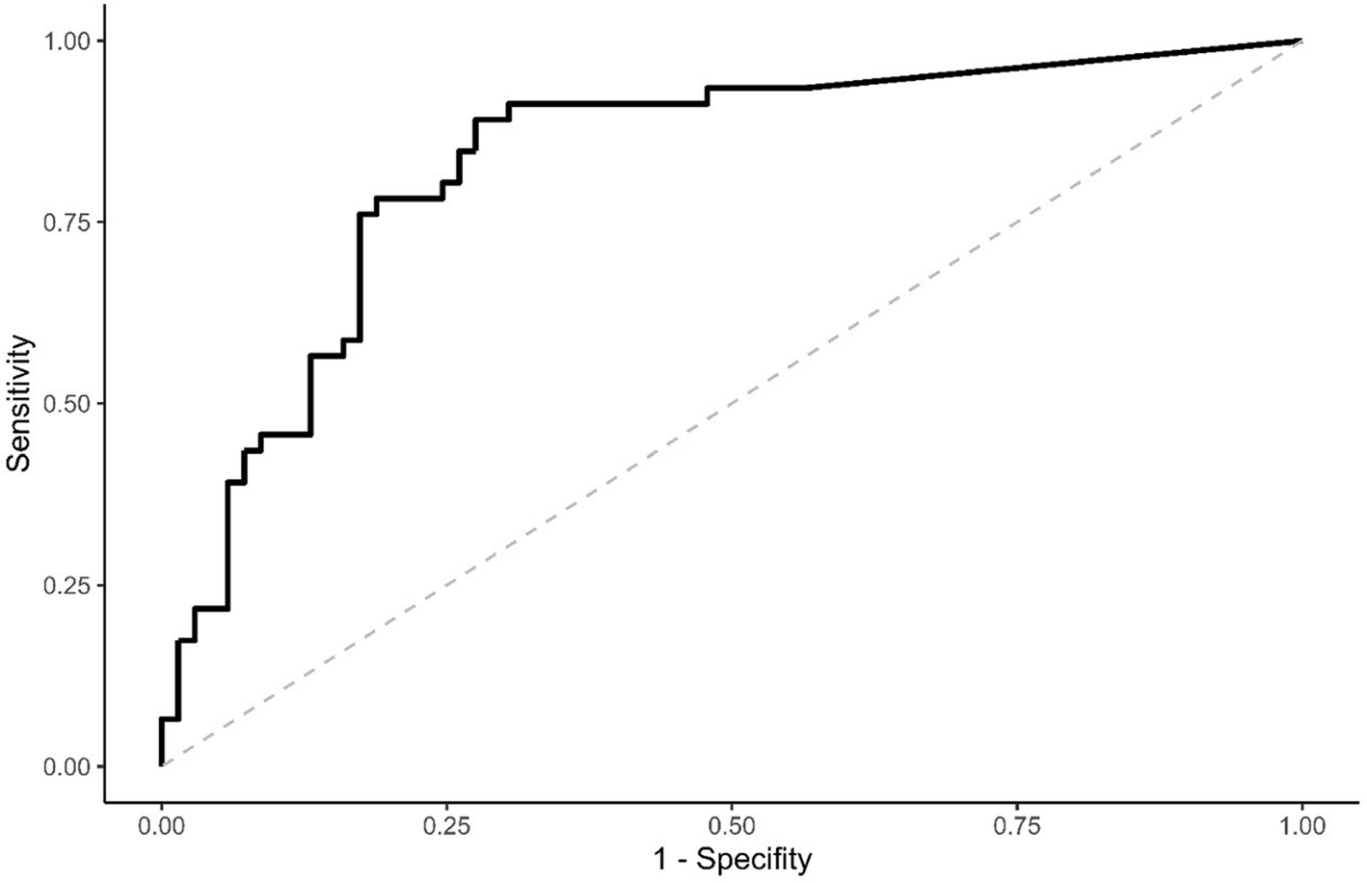
Receiver operating characteristic (ROC) curve for RA patients achieving low-disease activity or remission with LEF (DAS28-ESR ≤ 3.2) versus A77 1726 concentrations (n = 115). Low-disease activity or remission was defined if the patients achieved a DAS28-ESR ≤ 3.2 (low-disease activity or remission). Area under the curve = 0.83 (95% Confidence Interval; 0.76 to 0.91). Cut-off of A77 1726 concentrations was > 10.0 µg/mL, sensitivity, 91.31%; specificity, 73.91%.

Figure 1 shows the ROC curve for the DAS28-ESR response to serum levels of A77 1726. The area under the curve was 0.83 (95% confidence interval [CI], 0.76 to 0.91). We identified the best cut-off to classify RA patients as: (a) those who achieved low-disease activity or remission (DAS28-ESR ≤ 3.2); and (b) those who remained with moderate or severe disease activity despite the treatment (DAS28-ESR > 3.2) when treated with LEF at a serum concentration > 10.0 µg/mL A77 1726. This cut-off had a sensitivity of 91.1% and specificity of 72.5% to distinguish between RA patients who achieved low-disease activity or remission (DAS28-ESR ≤ 3.2) and those who remained with moderate or severe disease activity despite the treatment (DAS28-ESR > 3.2).

Table 2 shows the results of the correlation analysis between serum levels of A77 1726 and clinical variables. High serum levels of A77 1726 were correlated with low DAS28-ESR (*p* < 0.001). Correlations between serum levels of A77 1726 with age (*p* = 0.139) or disease duration (*p* = 0.099) were not detected.

**Table 2:** Correlations between serum A77 1726 levels and clinical and laboratory variables.

Variable n = 115	Serum levels of A77 1726	
	r	*p*
Age (years)	0.139	0.139
Disease duration (years)	0.155	0.099
Number of swollen joints (28 joints)	− 0.396	< 0.001
Number of tender joints (28 joints)	− 0.381	< 0.001
Pt. perception of morning stiffness (VAS 0–100 mm)	− 0.256	0.006
Pt. perception of pain (VAS 0–100 mm)	− 0.224	0.016
Pt. perception of severity of disease (VAS 0–100 mm)	− 0.460	< 0.001
DAS28-CRP	− 0.420	< 0.001
DAS28-ESR	− 0.421	< 0.001
C-reactive protein (mg/L)	− 0.232	0.013
Erythrocyte sedimentation rate (mm/h)	− 0.173	0.064

Correlations were obtained using Pearson correlation tests (r value).

Abbreviations: *Pt* patient, *VAS* visual analogue scale, *DAS28-CRP* disease activity score using C-reactive protein, *DAS28-ESR* disease activity score using erythrocyte sedimentation rate.

**Figure 2:**
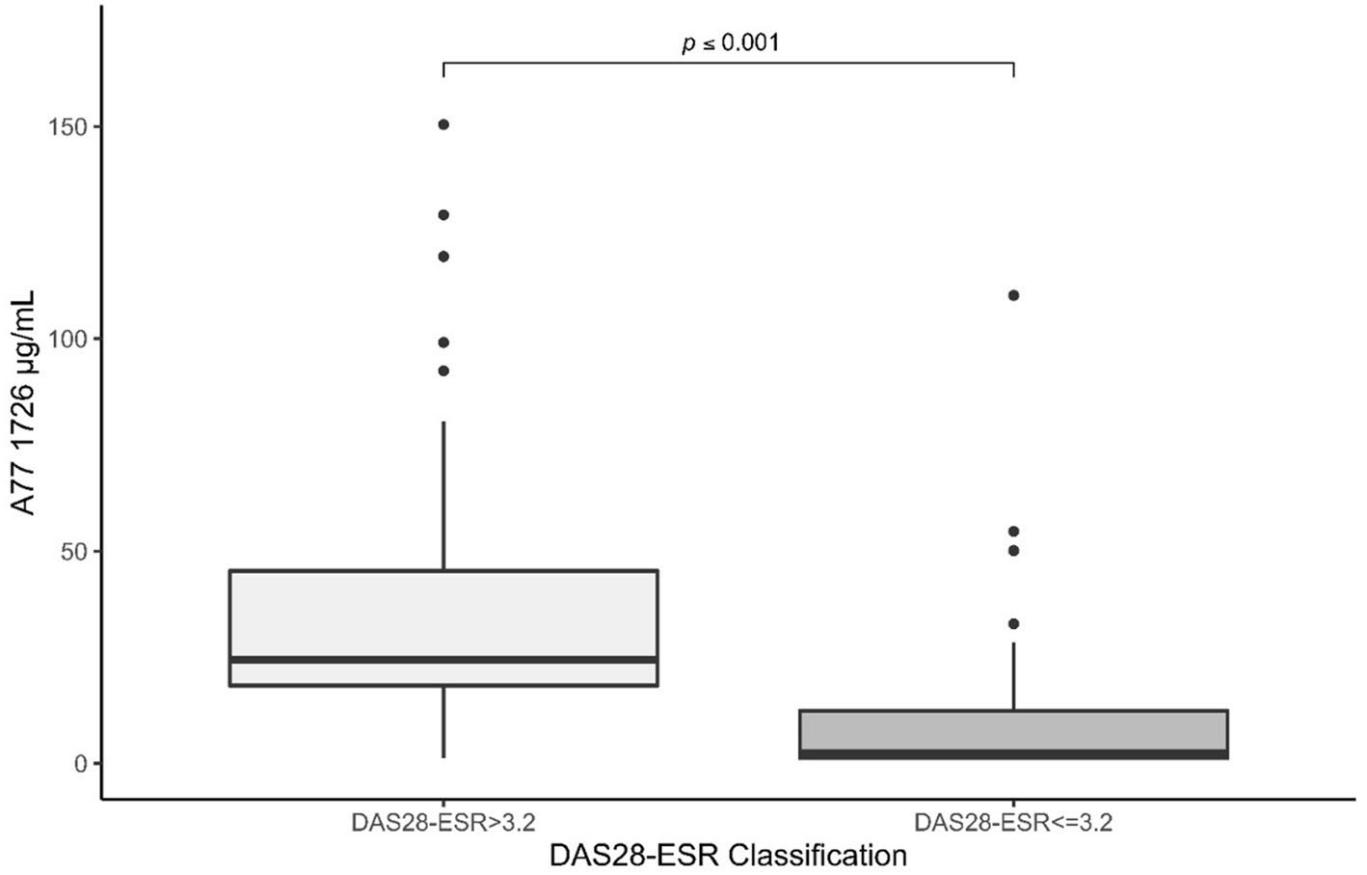
Box and whisker plots for A77 1726 serum concentrations in both groups, DAS28-ESR ≤ 3.2 and DAS28 > 3.2. Circles indicate outlying values.

Serum levels of A77 1726 varied greatly among RA patients who achieved low-disease activity or remission (DAS28-ESR ≤ 3.2), compared with those who remained with moderate or severe disease activity (DAS28-ESR > 3.2) despite the treatment with LEF; 25.21 [range: 1.24–119.36 µg/mL] versus 3.11 [range: 1.24–150.44]), respectively; *p* < 0.001) (Fig. 2).

In Table 3, using the cut-off point for A77 1726 > 10 µg/mL obtained from the ROC curve, we compared clinical and laboratory variables between RA patients with serum levels of A77 1726 ≤ 10.0 µg/mL and RA patients with serum levels of A77 1726 > 10.0 µg/mL. The results revealed that RA patients with A77 1726 > 10 µg/mL were older (*p* = 0.048), had fewer swollen and tender joints (*p* < 0.001), had less morning stiffness, pain, and perception of disease severity (*p* < 0.001), and had lower ESR and CRP (*p* = 0.036 and *p* < 0.001, respectively). These patients with serum levels of A77 1726 > 10.0 µg/mL presented lower disease activity, as measured by DAS2–-ESR (*p* < 0.001) and DAS28-CRP (*p* < 0.001).

**Table 3:** Comparison of characteristics between RA patients with serum A17 7726 concentrations ≤ 10 µg/mL versus RA patients with serum A17 7726 concentrations > 10 µg/mL.

Variable n = 115	A77 1726		*p*
	≤ 10 (µg/mL)	> 10 (µg/mL)	
Age (years)	57.5 ± 9.5	61.5 ± 12.0	0.048
Body mass index (kg/m^2^)	27.8 ± 3.7	27.3 ± 5.1	0.575
Disease duration (years)	14.3 ± 8.8	17.1 ± 12.9	0.173
Swollen joints (28 joints)	5.4 ± 3.3	0.9 ± 1.5	< 0.001
Tender joints (28 joints)	6.5 ± 4.0	1.5 ± 1.7	< 0.001
Pt. perception of morning stiffness (VAS 0–100 mm)	54.0 ± 25.7	31.6 ± 21.6	< 0.001
Pt. perception of pain (VAS 0–100 mm)	52.4 ± 29.1	32.6 ± 22.8	< 0.001
Pt. perception of severity of disease (VAS 0–100 mm)	71.4 ± 18.6	30.7 ± 23.9	< 0.001
Erythrocyte sedimentation rate (mm/h)	31.7 ± 9.7	27.6 ± 10.9	0.036
C-reactive protein (mg/L)	23.1 ± 17.7	12.4 ± 11.6	< 0.001
DAS28-CRP	5.0 ± 1.3	2.9 ± 1.0	< 0.001
DAS28-ESR	5.3 ± 1.1	3.3 ± 1.1	< 0.001
Corticosteroids doses (mg/day)	5.4 ± 5.3	4.1 ± 2.8	0.132

Comparisons of proportions between groups were computed using chi-square tests or Fisher’s exact test.

*Corticosteroid doses* in equivalence of prednisone, *ESR* erythrocyte sedimentation rate, *CRP* C-reactive protein, *VAS* visual analogue scale, *Pt* patient, *DAS28* disease activity score.

RA patients achieving low-disease activity or remission with LEF (DAS28-ESR ≤ 3.2) had higher serum levels of A77 1726 compared with those that remained with moderate/severe disease activity after treatment with LEF (34.50 ± 27.60 µg/mL and 14.10 ± 26.82 µg/mL respectively; *p* < 0.001). On the other hand, there was no difference between RA patients who achieved low-disease activity or remission (DAS28-ESR ≤ 3.2) and between age, BMI, RA disease duration, and corticosteroid dosage (data not depicted in a table).

A subgroup analysis including RA patients treated with LEF as monotherapy (n = 54) demonstrates that RA patients with serum levels of A77 1726 > 10 µg/mL (n = 27) had lower DAS28-ESR compared with those with serum levels of A77 1726 ≤ 10.0 µg/mL (n = 27) (3.0 ± 0.8 and 5.3 ± 1.1, respectively; *p* < 0.001). In the second subgroup analysis, we included RA patients treated with LEF as combo therapy with other DMARDs (n = 61). In this subgroup treated with LEF as combo therapy, RA patients with serum levels of A77 1726 > 10 µg/mL (n = 33) had lower DAS28-ESR compared with those with serum levels of A77 1726 ≤ 10.0 µg/mL (n = 28) (3.6 ± 1.2 and 5.3 ± 1.1, respectively; *p* < 0.001) (data not depicted in a table). These findings indicate that serum levels of A77 1726 constitute a good marker of therapeutic response, independently of whether LEF was prescribed as monotherapy or if it was prescribed in combination with other DMARDs.

The utility values in RA patients treated with LEF (serum levels of A77 1726 > 10 µg/mL to determine the probability of an adequate therapeutic response [DAS28-ESR ≤ 3.2]), are shown in Table 3. A high sensitivity (91.31%) was observed in RA patients treated with LEF used as monotherapy or in combination with other DMARDs. The specificity was moderate to high; and typically higher for those treated with LEF as monotherapy (80.64%). Moreover, the positive predictive value was clinically relevant for all patients treated with LEF (70.00%), both as monotherapy (77.78%), and combined with other DMARDs (63.64%). Nonetheless, a negative predictive value indicated that, if RA patients treated with LEF did not achieve serum levels of A77 1726 > 10 µg/mL, the probability of a lack of response to the medication was ~ 92%, independently of whether they were treated with LEF as monotherapy (92.59%) or with as combined therapy with other DMARDs (92.86%).

A77 1726 > 10 µg/mL was a protective factor against the permanence of a DAS28-ESR > 3.2 (OR: 0.034, 95% CI: 0.011 to 0.11, *p* < 0.001). To investigate the strength of the association between the different levels of A77 1726 and the risk of a lack of therapeutic response to LEF (DAS28-ESR > 3.2), we classified the following ranges of A77 1726 levels: (a) 1–4 µg/mL (n = 46), (b) 4.1–10 µg/mL (n = 9), (c) 10.1–30 µg/mL (n = 37), and (d) ≥ 30.1 µg/mL (n = 23). For this analysis we identified an increased risk of therapeutic failure when the lowest range of A77 1726 levels was compared to each interval of A77 1726 levels. When the lowest range (1–4 µg/mL) was compared to the 4.1–10 µg/mL range the risk of therapeutic failure was not different (OR = 1.3, 95% CI = 0.12–13.33, *p* = 0.8), but when the lowest range was compared to the 10.1–30 µg/mL range the risk of therapeutic failure in RA patients increased significantly in those with the lowest levels (OR = 7.2, 95% CI = 2.1–24.2, *p* < 0.001); finally, when the lowest range of A77 1726 levels was compared to > 30.1 µg/mL range the risk of therapeutic failure again saw a significant increase in RA patients with the lowest levels (OR = 16.3, 95% CI = 4.3–67.4, *p* < 0.001). In the comparison of the proportions of RA patients with lack of therapeutic response between the ranges of A77 1726 levels from 10.1 to 30 µg/mL to ≥ 30.1 µg/mL, no significant differences were observed (OR = 2.3, 95% CI = 0.8–6.6, *p* = 0.13), meaning that A77 1726 levels > 10 µg/mL are a good indicator of the therapeutic response to LEF.

## Discussion

This study demonstrates that high concentrations of A77 1726 have a relevant positive predictive value to RA patients who achieved low disease activity or remission (DAS28-ESR ≤ 3.2) when treated with LEF independently if LEF was used as monotherapy or as combo therapy with other DMARDs. We identified that higher concentrations of A77 1726 were correlated with a decrease in DAS28-ESR, as well as with a decrease in most parameters of disease activity. We identified that serum levels of A77 1726 > 10 µg/mL were a marker of RA patients who achieved low disease activity or remission with LEF (DAS28-ESR ≤ 3.2), with a sensitivity of 91.31% and specificity of 73.91%. However, in the present study, this cut-off was obtained using ROC curves. Although it is important to consider that the present study is cross-sectional, our findings do not reflect causality and should be considered as statistically demonstrated associations; therefore, further prospective longitudinal studies are mandatory to support our findings.

Moreover, we identified that higher serum levels of A77 1726 were correlated with a decrease in the number of swollen joints. These findings are supported by Chan et al., who observed a similar association between serum levels of A77 1726 and the number of swollen joints^13^, although, Chan et al. did not evaluate disease activity with DAS28-CRP or DAS28-ESR. In a cross-sectional study, van Roon identified that all RA patients who had serum levels of A77 1726 ≥ 16 mg/L achieved a good therapeutic response to LEF treatment^14^. Nevertheless, these studies only included patients with LEF treatment as monotherapy. In contrast, we included two subgroups: patients with LEF treatment as monotherapy and those with LEF treatment as combo therapy with other DMARDs. Therefore, we were able to demonstrate that high serum levels of A77 1726 were associated with the therapeutic response to LEF treatment, not only in monotherapy but also in patients treated with LEF as combo therapy. Wiese et al. also identified a relationship in 67 RA patients, between plasma A77 1726 levels and lower DAS28^15^. Similar to our study, Wiese et al. included two subgroups: (a) patients with a lack of response to LEF treatment with a combined therapy of methotrexate, sulfasalazine, and chloroquine; and (b) LEF treatment as monotherapy. However, these authors did not report the findings of these subgroups separately, which make interpretation of their results difficult. These researchers also identified an association between an increase in LEF doses and higher serum levels of A77 1726^15^.

In the present study, serum levels of A77 17226 > 10 µg/mL were associated with an increase in the therapeutic response to LEF, independent of whether LEF was used as monotherapy or as an additional antirheumatic drug in combo with other DMARDs in patients who did not achieve a good clinical response. In RA patients, A77 1726 exerts its action through the regulation of the immune response by modulating lymphocyte proliferation. A77 1726 inhibits the enzyme dihydroorotate dehydrogenase (DHODH), a key enzyme in the uridine monophosphate (rUMP) pathway^16,17^. Activated lymphocytes require approximately eightfold increased levels of rUMP to change from the G1 to the S phase of the cell cycle^18^. Inhibition of DHODH by A77 1726 arrests lymphocytes in the G1 phase of the cell cycle, thus decreasing lymphocyte proliferation and their proinflammatory immune response^19^.

As LEF is a pro-drug, one of the most relevant aspects in developing its relationship with clinical response is the quantity of the active metabolite, A77 1726, needed to exert its effects on joint inflammation.

In RA patients consuming LEF, there is interindividual variability in LEF absorption, distribution, and metabolism. This variation produces a large variability in serum levels of A77 1726 in a rheumatoid population, as described by van Roon et al. (3–150 mg/L)^14^, Grabar et al. (1.9–156.9 mg/L)^20^ and Chan et al. (11.8–101.5 mg/L)^13^. The large interindividual variability of serum levels of A77 1726 is, among others factors, a criterion to be considered for clinical pharmacokinetic monitoring^21^ as a tool for guiding therapy.

Furthermore, we observed in our RA patients that a cut-off of A77 1726 levels > 10 µg/mL was associated with a high sensitivity (91.31%) and moderate specificity (73.91%), to identify between RA patients with an adequate therapeutic response. van Roon et al.^14^ identified that at a serum level of 18 mg/L A77 1726, the patients presented a sensitivity of 90.9% for adequate therapeutic response, and at 16 mg/L A77 1726, the sensitivity was 100%. These findings support the sensitivity of 91% observed in our study, even though van Roon et al. did not investigate the differences between patients with LEF treatment as monotherapy and as combo therapy. Additionally, we observed that RA patients treated with LEF who had serum levels of A77 1726 > 10 µg/mL had a probability of 70% of achieving an adequate therapeutic response according to the positive predictive value; this probability was reduced to 63% in the subgroup of RA patients using LEF as combo therapy with other DMARDs. van Roon et al. observed a lower positive predictive value of 56% for serum levels of A77 1726 > 16 mg/L for obtaining an adequate therapeutic response with LEF treatment^14^. These findings support our observations regarding the clinical utility of measuring serum levels of A77 1726 in RA patients receiving treatment with LEF. Finally, we identified that RA patients treated with LEF with serum levels of A77 1726 ≤ 10 µg/mL had an approximately 92% probability of a lack of therapeutic response to treatment. This information can be relevant in expanding the clinical armamentarium used in clinical decision-making, as well as to generate future research questions related to the causes of low serum levels of A77 1726.

Additionally, we demonstrated the strength of association between the risk of therapeutic failure to LEF and low serum teriflunomide levels by comparing different ranges of A77 1726 levels. In this analysis, we identified the progression in the risk of therapeutic failure to LEF with the lowest ranges of A77 1726 levels when that range was compared to the strata of higher A77 1726 levels. This statistical analysis was not performed by any of the previous studies and constitutes a contribution of the present study by demonstrating the strength of the association between the different A77 1726 levels and the therapeutic response.

Our study has several limitations to be discussed. First, cross-sectional studies are unable to demonstrate causal relationships and their objectives are limited to identifying statistical associations that generate new questions and hypotheses that require demonstration in further longitudinal designs. In the same sense, the associations observed between nonresponse to leflunomide and the low teriflunomide levels identified in our study cannot be interpreted as demonstration of causality. However, this cross-sectional design is a relevant approach for evaluating the role of high teriflunomide levels as a diagnostic test in RA patients with treatment failure to leflunomide. The results of the utility values of high teriflunomide levels based on the cut-off of A77 1726 > 10 µg/mL require further validation by new similar studies in other populations. Second, another limitation of our study is that we did not assess the role of CYP2C19 variant statuses in RA patients in its results. CYP2C19 variant statuses can influence A77 1726 levels and subsequently the therapeutic response in RA patients^22^. Therefore, further studies should include an assessment of CYP2C19 variants and teriflunomide levels in the same study. An important issue to be considered in the discussion of the findings of this study is that there is evidence regarding the influence of inflammation in downregulating the activity of CYPC219, through the induction of epigenetic modifications of drug metabolism enzymes and transporter (DMET) genes, which lead to a decrease in the level of DNA methylation and a reduced expression and low function of CYP2C19^23^. Inflammation is associated with the expression and upregulation of miRNAs that decrease DMET expression, block gene transcription and downregulate CYP2C19^24,25^. Therefore, an explanation of the low levels of teriflunomide in the nonresponders patients observed in our study can be attributed to the chronic inflammation mediated effects on the phenotypic changes of the drug metabolizing enzyme CYP2C19, which recategorizes these patients from being extensive metabolizers to poor metabolizers^26^. In this context, the correlations observed in our study between low teriflunomide levels and high DAS28-ESR, and DAS28-CRP and CRP levels could be explained by the effects of chronic inflammation decreasing the function of CYP2C19. This hypothesis must be corroborated by new studies that simultaneously assess teriflunomide levels and the activity of the CYPC2C19 enzyme.

Finally, one of the main criticisms of cross-sectional designs is that they cannot demonstrate temporal relationships between exposure and events. This critique also applies to the present study; therefore, although this cross-sectional study is not able to demonstrate that the serum teriflunomide concentrations observed in our patients were prior to the therapeutic response, the hypothesis that these A77 1726 levels can be a relevant factor in obtaining an adequate response can be supported by other relevant criteria, as described by Bradford Hill^27^. First, we demonstrated the strength of the association between A77 1726 levels and the DAS28-ESR results, which identifies a statistical correlation between these variables. Second, the following criteria supporting the hypothesis of A44 1726 levels as a factor of therapeutic response are consistent with the findings of other studies. We have previously discussed the evidence of the studies performed by Chan et al., van Roon et al. and Wiese et al.^13–15^ which support a cut-off of A77 1726 levels in identifying RA patients with a therapeutic response to LEF. Third, we also demonstrated a biological gradient between lower A77 1726 levels and the increasing risk of a lack of therapeutic response. Fourth, there is also a substantive evidence of biological plausibility to consider that A77 1726 levels are factors in the therapeutic response and not a mere consequence of the inflammation. This evidence is derived from experimental studies and clinical trials; Thoss et al. using a rat antigen-induced arthritis model demonstrated an increase on the response of the knee inflammation after the administration of incremental doses of LEF^28^; whereas, Cherwinski et al. demonstrated differences in the half-maximal inhibitory concentration (IC50) for the proliferation of mitogen-stimulated lymphocytes of rats, mice and humans; which is dependent on the A77 1726 concentration^29^. In the same sense, early randomized controlled clinical trials using LEF have identified dose response as one of the factors implied in the benefits obtained with this drug by RA patients^30^. Another criterion that supports our results is their coherence with the findings observed by other investigators. Weber et al. calculated the mean serum A77 1726 levels and the proportions of patients achieving a steady state and obtained a concentration of 13 mg/L (µg/mL) as cut-off for that they denominated as an “optimal clinical success”^14,31^. In a recent experimental study, Wostradowski, et al. demonstrated that teriflunomide significantly downregulated the surface expression of the costimulatory molecule CD86 in a concentration- and time-dependent manner in rat microglia^32^. This CD86 receptor is necessary for the double and simultaneous interaction between antigen-presenting cells (APCs) and T lymphocytes, which is included in the pathways of the immunological response increased in RA^33,34^. On the basis of all these criteria but lacking the most important causality criterion—temporality—we consider that there is substantive evidence for considering that A77 1726 levels have relevance in the therapeutic response of RA patients treated with LEF; however, our findings only demonstrated relevant statistical associations but not causality and further longitudinal studies should be performed to demonstrate any potential causal relation between teriflunomide levels and therapeutic outcomes.

## Conclusions

This study identified that serum levels of A77 1726 > 10 µg/mL were a good clinical tool for predicting therapeutic response, not only in RA patients using LEF as monotherapy but also in RA patients who failed to respond to other DMARDs and had LEF included as combo therapy to improve therapeutic response. Therefore, we propose that the measurement of serum levels of A77 1726 should be considered a useful tool in guiding treatment modifications in RA patients treated with LEF.

## Materials and methods

### Study design

Cross-sectional study.

### Clinical setting

This study included adult patients with RA attending an Academic Research Center (Instituto de Terapéutica Experimental y Clínica, Departamento de Fisiología Centro Universitario de Ciencias de la Salud, Universidad de Guadalajara, México).

### Subjects and methods

We included RA patients who met the 1987 American College of Rheumatology (ACR) criteria^35^, ≥ 18 years old, who started LEF treatment as a consequence of the presence of moderate to severe disease activity despite treatment with methotrexate or other csDMARDs. RA patients treated with LEF were divided into two groups according to their treatment: (a) LEF used as monotherapy (without other csDMARDs), or (b) LEF used as combo therapy with other csDMARDs. All the RA patients in our centre that were candidates to receive this drug were instructed to receive LEF as one single tablet of 20 mg administered once daily (preferably after breakfast). Patients from both groups had to be treated with LEF at a stable dosage (20 mg/Day P.O.) for a period of at least three months to a maximum of six months to evaluate the therapeutic response. We excluded pregnant or breastfeeding patients, patients with serum creatinine > 1.5, transaminases > twofold normal values, or with acute or chronic infections. We also excluded patients with overlapping syndromes (i.e., characteristics of an additional autoimmune connective tissue disease present in the same patient, including systemic lupus erythematosus, scleroderma, poly or dermatomyositis, etc.).

### Clinical assessment

A structured assessment of patients with RA was performed by two researchers; this assessment included a structured questionnaire of epidemiological variables (including age, smoking, and other characteristics), a physical examination and body mass index (BMI) calculation(—using the Quetelet formula (W/H^2^), weight (W) and height (H [at square]—))^36^, a review of disease characteristics, patients’ perception of morning stiffness, severity of pain, and the perception of disease severity using a visual analogue scale from 0 to 100 mm (VAS 0–100 mm). This evaluation also included the number of tender joints and swollen joints (28 joints evaluated in total).

To assessment of disease activity, we included the Disease Activity Score (DAS28), which is a pooled index that identifies the severity of disease activity and includes four items: (a) the number of tender joints, (b) the number of swollen joints, (c) patients’ perception of global health (from 0 meaning very well to 10 meaning very poor), and (d) the results of acute-phase reactants (alternative erythrocyte sedimentation rate [ESR] or C-reactive protein [CRP]). These data were included in a formula to calculate the DAS28 score^37^; DAS28-ESR and DAS28-CRP were also computed^38–40^.

### Assessment of therapeutic response

DAS28-ESR was assessed at three to six months of treatment with LEF^41^. The primary target should achieve a state of clinical remission or at least clinical low disease activity, according to the patient’s therapeutic goals^42^. Therefore, we classified the response to LEF in our patients into two groups (given by the DAS28-ESR achieved during the second visit to the rheumatologist after 3–6 months of prescribing LEF): (a) those who achieved low-disease activity or remission (DAS28-ESR ≤ 3.2); and (b) those who remained with moderate or severe disease activity despite the treatment (DAS28-ESR > 3.2).

Finally, the researchers performed a chart review to investigate the ongoing drugs and doses used. The assessment of LEF doses was made by identifying the doses prescribed by the rheumatologist and cross-checking against a questionnaire of adherence designed specifically for this study before applying it to RA patients. Researchers also investigated whether the patients were taking other csDMARDs and the name of other DMARDs prescribed, and then classified the LEF prescription in two groups: (a) LEF as monotherapy and (b) LEF used as combo therapy in those patients who did not achieve a DAS28-ESR ≤ 3.2 using other csDMARDs. Patients were also interviewed to verify whether they had withdrawals from any medications.

### Quantification of acute-phase reactants

ESR was determined using the Westergren method, and the results were expressed in mm/h. CRP was determined using nephelometry, and the results were expressed in mg/L.

### Serum determination of LEF

Nonfasting blood samples were taken by venipuncture in all patients during the early morning hours. Each blood sample was centrifuged immediately to obtain the serum, which was stored in Eppendorf tubes at − 80 °C until the A77 1226 levels were quantified. Serum samples were analysed to quantify A77 1726 levels by a validated high-pressure liquid chromatographic method, using an Agilent 1260 UHPLC (Santa Clara, California, United States of America). Separation of the analytes was accomplished using a reversed-phase column (Phenomenex, C18, 4µ, 150 × 4.6 mm; Torrens, California, United States of America). The column temperature was maintained at 21 °C. The mobile phase consisted of 40 mM (pH 5.0) KH_2_PO_4_:methanol (1:1). Elution of A77 1726 was achieved at a flow rate of 1.2 mL/min. UV detection was set at 290 nm.

### Statistical analysis

Quantitative variables were expressed as means and standard deviations, and qualitative variables, as frequencies and percentages (%). We used the Pearson correlation test to verify whether there was correlation between the serum levels of A77 1726 and swollen joints. Receiver operator characteristics (ROC) curves were used to determine the cut-off of A77 1726 concentrations with better performance in sensitivity and specificity to identify patients with low disease activity or remission and differentiate them from patients with moderate or severe disease activity according to the DAS28-ESR results. RA subjects were categorized into two groups, according to therapeutic response: (a) Group 1, for RA patients who achieved low-disease activity or remission with LEF (DAS28-ESR ≤ 3.2); and Group 2, for RA patients who remained with moderate/severe disease activity after the treatment (DAS28-ESR > 3.2). Additionally, we performed a stratified analysis of two subgroups: the first subgroup included RA patients receiving LEF as monotherapy, and the second included RA patients receiving LEF as combo therapy with other DMARDs due to lack of response to previous treatments. The performance of the measurements of A77 1726 levels at the cutoff obtained by the ROC curve analysis, to identify low-disease activity or remission with LEF in patients with RA was evaluated by estimating sensitivity, specificity, and positive and negative predictive values.

Comparisons of proportions between RA patients with A77 1726 concentrations ≤ 10 µg/mL and A77 1726 concentrations > 10 µg/mL were computed using Chi-square tests (or Fisher exact test if required), and comparisons between means were made using Student’s t test). A similar statistical approach was used in the comparison between RA patients who achieved low-disease activity or remission with LEF (DAS28-ESR ≤ 3.2) and RA patients who remained with moderate to severe disease activity after the treatment (DAS28-ESR > 3.2) to LEF. We computed the odds ratio and its 95% confidence interval to identify the protective factor of A77 1726 > 10 µg/mL to the permanence of moderate/severe disease activity (DAS28-ESR ≤ 3.2). Data collection and statistical analyses were computed with R software version 4.0.0 for Windows (R core team 2020), using pRoc, epiR, and ggplot2 packages^43–45^. The *p* value was considered significant if *p* < 0.05.

### Ethics approval

The study protocol was approved by the Ethics Board of our Research Centre (Instituto de Terapeutica Experimental y Clinica, Universidad de Guadalajara, in Guadalajara, Mexico). Number of approval CEI/453/2018. All procedures in the protocol were performed according to the guidelines of the Declaration of Helsinki.

### Consent to participate

Informed consent was obtained from all individual participants included in the study.

## Supplementary Information

Supplementary Information.

## Data Availability

The datasets used and/or analysed during the current study are available from the corresponding author on reasonable request.
