# Supplementary material for: Therapeutic response to leflunomide in combo therapy and monotherapy is associated to serum teriflunomide (A77 1726) levels
Source: Sci Rep. 2022 Feb 3;12:1877. doi: 10.1038/s41598-022-05644-7 (PMC8814146; doi:10.1038/s41598-022-05644-7)
Supplement: Supplementary file 1 — Supplementary Information. [file 41598_2022_5644_MOESM1_ESM.docx]

**Consortia: Research Group for Factors Related to Therapeutic Outcomes in Autoimmune Diseases Affiliations**

**Senior researchers**

Gamez-Nava Jorge Ivan, Gonzalez-Lopez Laura, Leaders of the group, **Departamento de Fisiología, Programa de Doctorado en Farmacología and Programa de Doctorado en Salud Publica Centro Universitario de Ciencias de la Salud, Universidad de Guadalajara**; Cardona-Muñoz Ernesto German, **Centro Universitario de Ciencias de la Salud, Departamento de Fisiología, Universidad de Guadalajara**.

**Associated Researchers**

**Research in Clinical and Laboratory Analyses** Fajardo-Robledo Nicte Selene, **Centro Universitario de Ciencias Exactas e Ingenierías, Laboratorio de Investigación y Desarrollo Farmacéutico, Universidad de Guadalajara;** Saldaña-Cruz Ana Miriam, Rodriguez-Jimenez Norma Alejandra, Ramirez-Villafaña Melissa, **Centro Universitario de Ciencias de la Salud, Departamento de Fisiología, Universidad de Guadalajara**; Nava-Valdivia Cesar Arturo, **Departamento de Microbiologia y Patologia, Centro Universitario de Ciencias de la Salud, Universidad de Guadalajara;** Ponce-Guarneros Juan Manuel, **Centro Universitario de Ciencias de la Salud, Departamento de Fisiología, Universidad de Guadalajara** and **Instituto Mexicano del Seguro Social, UMF 97, Guadalajara, Jalisco, Mexico**; Alcaraz-Lopez Miriam Fabiola, **Instituto Mexicano del Seguro Social, HGR 46, Guadalajara, Jalisco, Mexico.**

**Statistical Team**

Gamez-Nava Jorge Ivan, **Departamento de Fisiología, Programa de Doctorado en Farmacología and Programa de Doctorado en Salud Publica Centro Universitario de Ciencias de la Salud, Universidad de Guadalajara;** Alfredo Celis, **Departamento de Salud Publica Centro Universitario de Ciencias de la Salud, Universidad de Guadalajara.**

**Research Fellows**

Jacobo-Cuevas Heriberto, Olivas-Flores Eva, Gonzalez-Ponce Fabiola, Gomez-Ramirez Eli

**Centro Universitario de Ciencias de la Salud, Programa de Doctorado en Farmacología, Universidad de Guadalajara**
